# Elastic net regularized regression for time-series analysis of plasma metabolome stability under sub-optimal freezing condition

**DOI:** 10.1038/s41598-018-21851-7

**Published:** 2018-02-26

**Authors:** Gerard Bryan Gonzales, Sarah De Saeger

**Affiliations:** 1 Gastroenterology and Hepatology, Department of Internal Medicine, Faculty of Medicine and Health Sciences, Ghent University, C. Heymanslaan 10, 9000 Ghent, Belgium; 2 Laboratory of Food Analysis, Department of Bioanalysis, Faculty of Pharmaceutical Sciences, Ghent University, Ottergemsesteenweg 460, 9000 Ghent, Belgium

## Abstract

In this paper, the stability of the plasma metabolome at −20 °C for up to 30 days was evaluated using liquid chromatography-high resolution mass spectrometric metabolomics analysis. To follow the time-series deterioration of the plasma metabolome, the use of an elastic net regularized regression model for the prediction of storage time at −20 °C based on the plasma metabolomic profile, and the selection and ranking of metabolites with high temporal changes was demonstrated using the glmnet package in R. Out of 1229 (positive mode) and 1483 (negative mode) metabolite features, the elastic net model extracted 32 metabolites of interest in both positive and negative modes. L-gamma-glutamyl-L-(iso)leucine (tentative identification) was found to have the highest time-dependent change and significantly increased proportionally to the storage time of plasma at −20 °C (R^2^ = 0.6378 [positive mode], R^2^ = 0.7893 [negative mode], p-value < 0.00001). Based on the temporal profiles of the extracted metabolites by the model, results show only minimal deterioration of the plasma metabolome at −20 °C up to 1 month. However, majority of the changes appeared at around 12–15 days of storage. This allows scientists to better plan logistics and storage strategies for samples obtained from low-resource settings, where −80 °C storage is not guaranteed.

## Introduction

The importance of pre-analytical processes on blood samples used for subsequent metabolomics analyses has been thoroughly investigated in the past^1^. Strict observance of pre-analytical protocols, especially storage of samples, is necessary since highly dynamic and pronounced changes in the metabolome occurs as soon as the blood samples are drawn from the patients and continue as the samples are being prepared for metabolomics analysis. The instability of the plasma metabolome under different storage conditions has been previously reported, and there is a general consensus that an acceptable storage temperature for these biological samples should be −80 °C, especially for long-term storage^1–3^. For short-term storage, several reports agree that storage of plasma at −20 °C for one week does not cause significant deterioration of the plasma metabolome^2,3^.

This ultralow temperature requirement however presents a problem when the source of the biological material is a low-resource area, where a certified −80 °C storage and logistics cannot be guaranteed. Hence, as metabolomics is now being used as a powerful tool to investigate neglected tropical diseases^4^, it is useful to follow the slow deterioration of the plasma metabolome under sub-optimal freezing conditions, such as at −20 °C, in order to have a better estimate of how long these biological materials can be stored under limited resources for subsequent metabolomics analysis in a more equipped laboratory. This information will help logisticians to plan a better product flow strategy that is a good compromise between sample quality and resource limitations.

Data analysis of plasma metabolome stability in a time series often involves univariate analysis to determine the number of significantly different metabolites that changed across the entire time series. However, as this is univariate, interaction of 2 or more metabolites, that may not be statistically different individually, will not be determined. For time-series data, several methods have been proposed such as COVAIN toolbox (implemented in MATLAB©)^5^, partial least squares discriminant analysis (PLS-DA)^6^, and multivariate empirical Bayes statistical time-series analysis (MEBA)^7^. MEBA for instance, has been increasingly used as it is now integrated in the web-based easy-to-use platform, MetaboAnalyst^8,9^. MEBA is a time-course analysis method based on multivariate empirical Bayes statistic which could evaluate the importance of the temporal changes of metabolites using the Hotelling’s *T*^2^. The outcome is a ranked list of all metabolites that show differences in their temporal profile^7^. Metabolites with higher Hotelling’s *T*^2^ value comprise those whose profiles are more different across the time series. As this method ranks all metabolites, it is difficult to determine which Hotelling’s *T*^2^ value indicates that the particular metabolite is substantially changing from those whose temporal change is negligible.

In this paper, we describe a computational approach for the prediction of storage time of plasma at a sub-optimal freezing condition (−20 °C) based on the metabolome profile, which simultaneously ranks metabolites based on their model contribution while removing metabolites whose temporal changes are negligible through the entire time series using elastic net regularized regression. Elastic net is a generalization of the ridge regression and least absolute shrinkage and selection operator (LASSO)^10^. The combination of ridge and LASSO performs feature selection and handles multicollinearity within the dataset^11^, which are important characteristics for analysing datasets with large numbers of features (many of which could be collinear) and relatively smaller number of observations typical of omics studies^12^.

## Results

### Data pre-processing

LC-MS data were pre-processed using Progenesis QI. The processing resulted to the deconvolution and integration of 6749 and 5168 features in positive and negative modes, respectively. Progenesis QI uses both isotope and adducts deconvolution algorithm that identifies the parent ion and integrates the detected adducts (Na, K) into one feature. After filtering of the features based on QC dilutions correlation, %CV in QC samples and number of missing values, the final number of features amounted to 1229 and 1483 for positive and negative modes, respectively. These filtered datasets were then imported to MetaboAnalyst for further data processing and analysis.

**Table 1:** One-way repeated measures ANOVA of features through the time series.

t_R_ (mins)	m/z	F-value	FDR-adjusted *p*-value
*Positive mode*			
1.53	261.1414	21.3	1.26E-16
3.89	299.1453	9.9312	3.16E-08
3.79	301.1177	7.7681	3.08E-06
1.70	86.0963	6.727	3.06E-05
3.41	194.0811	6.5038	4.32E-05
0.69	230.0988	6.2241	6.68E-05
0.94	225.0735	6.2054	6.68E-05
0.40	298.0524	5.4046	4.86E-04
3.83	470.3217	4.8864	0.001
0.31	414.8628	3.6835	0.046
*Negative mode*			
1.64	260.1360^n^	37.859	7.78E-25
3.24	584.2636^n^	5.0645	0.0058421

^n^Neutral mass after adduct deconvolution.

### Univariate analysis

One-way repeated measures ANOVA revealed that 10 and 2 features significantly changed throughout the entire time series in positive mode and negative modes, respectively (Table 1). These features comprised of both water soluble and hydrophobic metabolites as evinced by their retention times through an HSS T3 C_18_ column, which was selected as it affords the retention of both hydrophilic and hydrophobic compounds^13^.

**Table 2:** Mobile phase and flow rate gradient parameters.

Mode	Time (min)	Flow rate (mL min^−1^)	%B	Gradient curve	Mode	Time (min)	Flow rate (mL min^−1^)	%B	Gradient curve
Positive	0	0.2	5	Initial	Negative	0	0.2	5	Initial
	1	0.2	8	10		3	0.3	50	8
	2	0.25	15	9		6	0.3	70	6
	4	0.25	60	8		7	0.35	100	6
	6	0.3	70	6		8	0.4	100	1
	7	0.35	100	6		8.5	0.4	100	1
	8	0.4	100	1		9	0.35	5	6
	8.5	0.4	100	1		10	0.2	5	1
	9	0.35	5	6					
	10	0.2	5	1					

**Figure 1:**
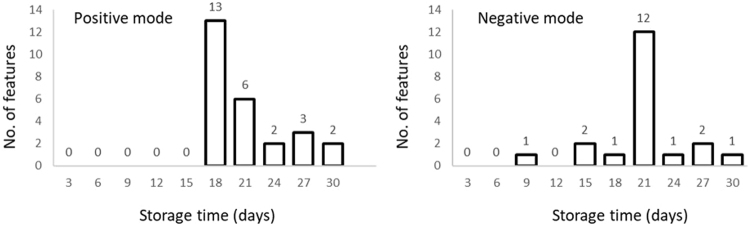
Evolution of number of features significantly (FDR p-value < 0.05) different across the time-series by pairwise comparison (paired t-test) with day 0 in both positive (left) and negative (right) modes.

By using a pair-wise comparison, the number of significantly different metabolite features in all time points were relatively low compared to day 0, with the maximum number of 13/1229 significantly different features appearing at day 18 in positive mode and 12/1483 significantly different features appearing at day 21 in negative mode (Fig. 1). Overall, the data suggests that plasma is relatively stable at −20 °C up to a month with very minimal degradation. A spiked increase in significantly different features at day 18 (positive mode) and 21 (negative mode) is curious and the reason is unknown. However, a similar pattern was reported for the metabolome stability of urine at −20 °C^14^.

### Multivariate empirical Bayes ANOVA (MEBA)

To determine the features/metabolites whose profile changed throughout the time series, data was first analyzed using the multivariate empirical Bayes ANOVA^7^ (MEBA) implemented in MetaboAnalyst^8,15^.

**Figure 2:**
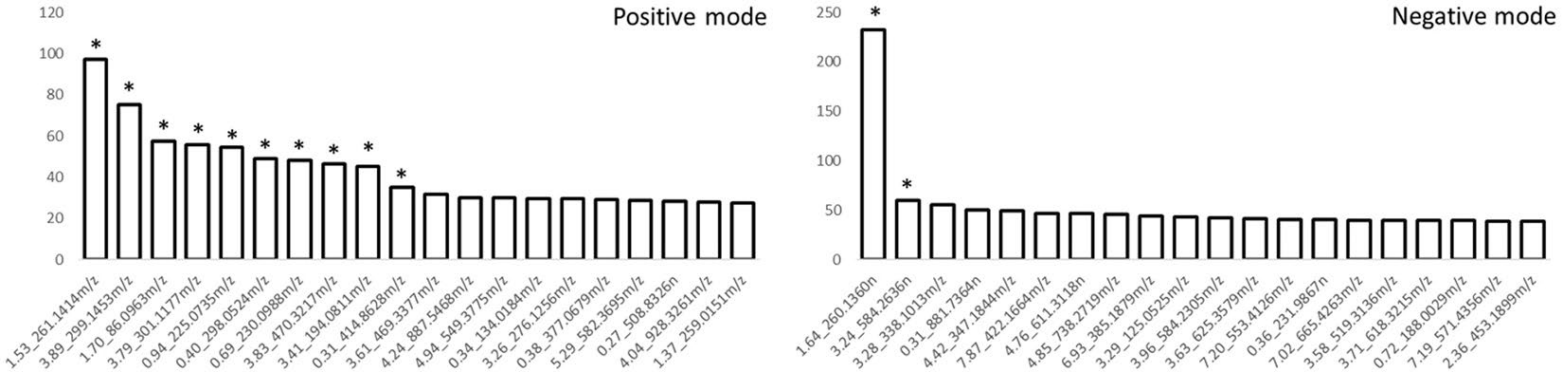
Hotelling’s *T*^2^ values of the top 20 features ranked using MEBA implemented using MetaboAnalyst. *Denotes significant differences over the entire time course (one-way repeated measures ANOVA, FDR p < 0.05).

As shown in Fig. 2, highly ranked metabolites in terms of Hotelling’s *T*^2^ statistics also showed significant differences over the entire time-course using repeated measures ANOVA. Considering that MEBA is in fact an extension of ANOVA, this result is therefore expected. The metabolite with a neutral mass of 260.1360 Da ranked highest in both positive and negative modes.

### Elastic net regularized regression model

As MEBA gives a rank to all metabolites detected, it is difficult to estimate which Hotelling’s *T*^2^ value indicates that the metabolites are actively changing through the time series. Hence, in this paper, we propose a method which ranks metabolites according to their temporal profile and also eliminates metabolites whose temporal change are not large enough to affect the metabolome significantly. The main assumption is that if the plasma metabolome changes during storage at −20 °C in a particular fashion, it is possible to predict the number of days that the plasma has been stored at −20 °C based on the metabolome profile. In this case, it is expected that a deteriorating metabolite would gradually decrease over time and a breakdown product is expected to gradually increase over time. Thus, an elastic net regularized regression model, which is a combination of the ridge regression and LASSO, was employed. Using this method, a feature selection strategy has been implemented that provides a list of metabolites that are predictive of storage time and ranks them based on their contribution to the model and consequently based on their temporal change.

**Figure 3:**
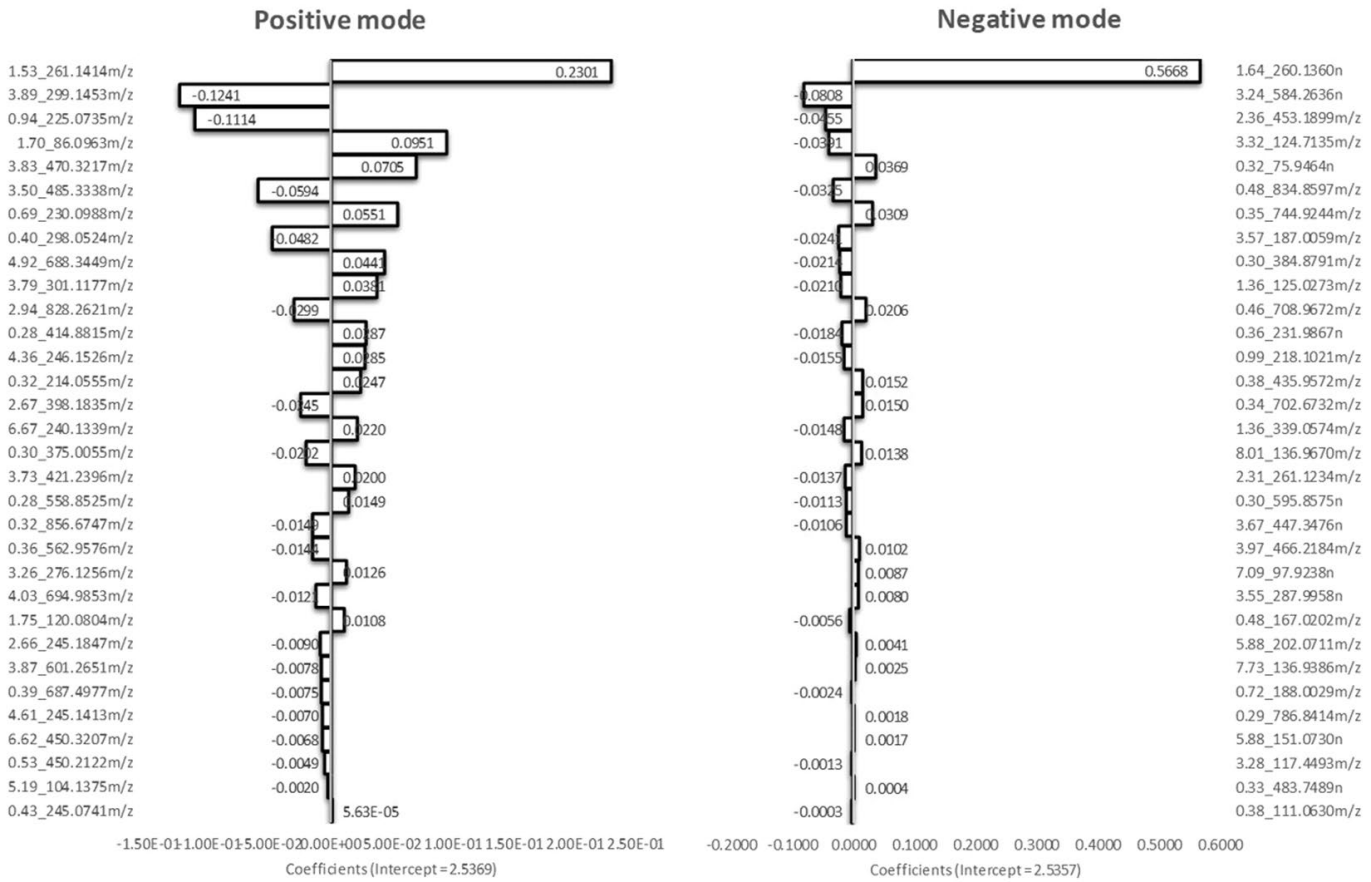
Model coefficients of metabolites extracted by elastic net regularized regression in both positive (left) and negative (right) modes. Higher absolute value of the coefficient reflect higher contribution to the model.

To build the model, a range of α values (0, 0.25, 0.5, 0.75, 1) was initially screened in order to select the best model, which could yield the lowest error. In the end, α = 0.5 was chosen, which gives equal weight to ridge regression and LASSO. In this case, we benefit both from the feature selection capability of LASSO and the ability of ridge regression to handle multicollinearity in the dataset. The optimum λ parameter on the other hand was determined based on cross-validated MSE. Figure 3 shows the metabolites extracted by the elastic net model along with their coefficients. Metabolites with higher absolute value of their coefficients signify higher contribution to the model predicting storage time. From 1229 metabolites detected in positive mode and 1483 metabolites in negative mode, the elastic net model was able to reduce the dataset into 32 metabolites in both positive and negative modes. The metabolites extracted by the elastic net model also ranked relatively high in MEBA, although not all of the top ranked metabolites of MEBA were extracted by the elastic net model. These metabolites are those with erratic changes in time and whose increase nor decrease does not significantly correlate with storage time. For example, metabolite feature 0.31_414.8628 (rt_m/z) ranked 10^th^ based on MEBA and was found to be statistically different over time based on ANOVA (FDR p-value = 0.0460). However, as its temporal change is erratic do not seem to follow a certain increasing or decreasing pattern (R^2^ = 0.0001, p = 0.91), metabolites as such may not be considered as a biomarker to assess or predict plasma degradation during storage at sub-optimal freezing conditions.

**Figure 4:**
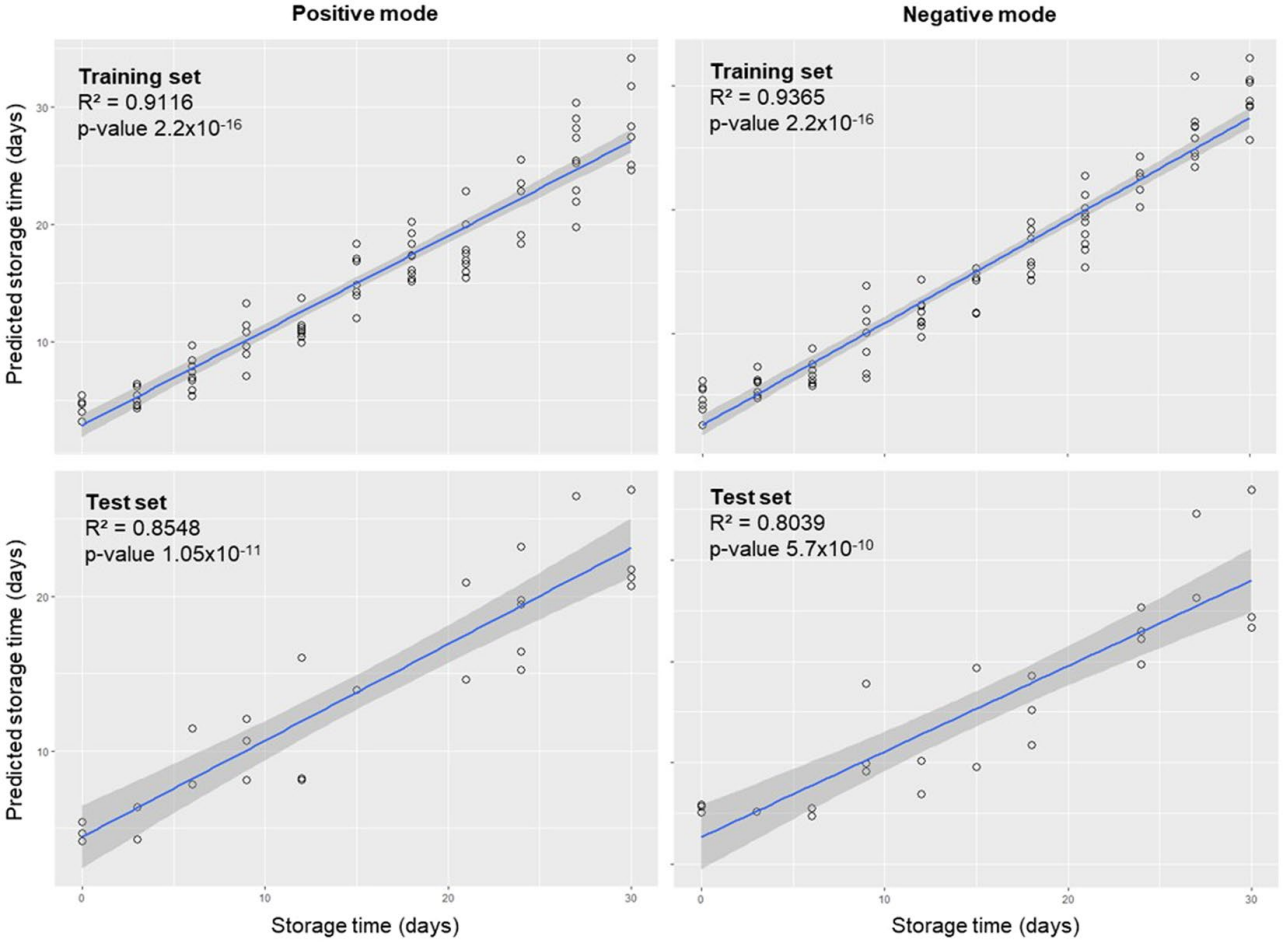
Correlation between experimental storage time of plasma at −20 °C versus predicted storage time by the elastic net regularized Poisson regression model in positive (left) and negative modes (right) in both training (top) and test (bottom) sets. Data were randomly split into training (75%) and test (25%), wherein the model was generated using the training set and validated using the test set.

Strong significant correlations (Fig. 4) were found between the actual storage times and predicted storage times in both positive and negative ionization modes, and both training and test sets. This indicates that the models are highly predictive. However, considering that elastic net imposes a constraint to the coefficients, no statistical significance (p-value) can be provided since the standards errors cannot be calculated from biased estimators. Instead of calculating the standard errors for biased coefficients, bootstrapping was performed during cross validation to assess the reproducibility of the results^16^. Several iterations of the model with 10-fold cross validation yielded the same metabolite features, which indicate the robustness of the selected metabolites for the prediction of storage time at −20 °C conditions.

**Figure 5:**
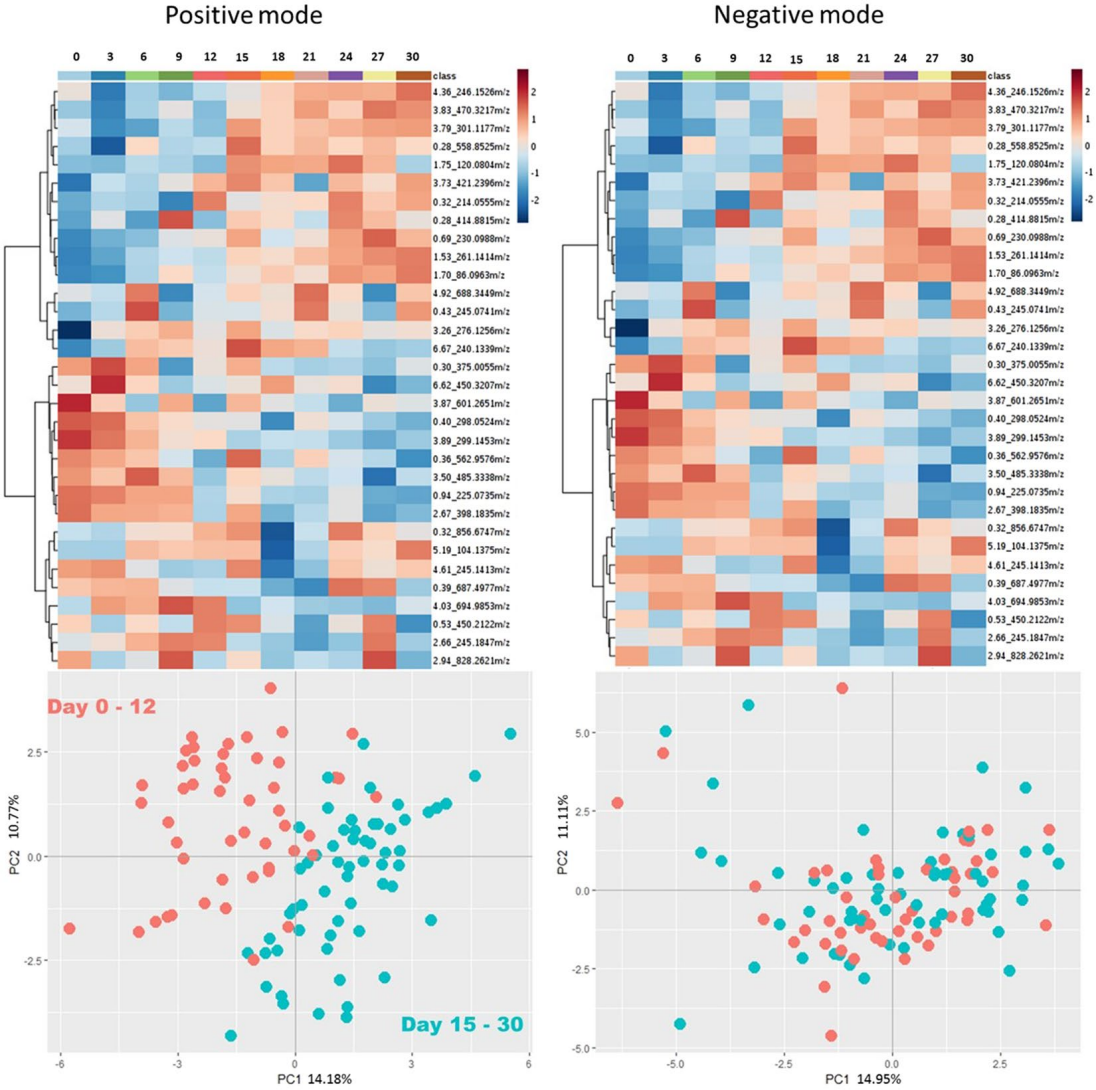
Heat maps (top) and PCA plots (bottom) of metabolites extracted by the elastic net regularized Poisson regression (glmnet) model over the time series in both positive and negative modes. The elastic net model extracted 32 metabolites of interest in both ionization modes.

Focusing on these extracted metabolites, a heat map shown in Fig. 5 graphically represents the degradation pattern of the plasma metabolome across the time series (30 days) at −20 °C. Using principal components analysis (PCA) for data visualization, it can be seen that there is a clear separation between samples stored from 0–12 days and those stored from 15–30 days in positive mode. In negative mode however, there is no clear-cut separation based on storage time. Nonetheless, the heat map (for negative mode) suggests that, similar to positive mode, more drastic changes in metabolite averaged intensities are observed at around 12–15 days of storage.

**Figure 6:**
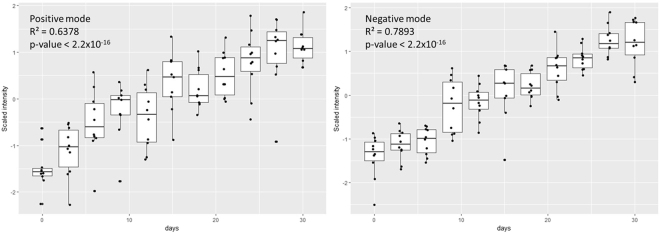
Correlation between the level of L-gamma-glutamyl-L-(iso)leucine and days of storage at −20 °C. Signal intensity of L-gamma-glutamyl-L-(iso)leucine increasing significantly proportional to storage time time (R^2^ = 0.6378 [positive mode], R^2^ = 0.7893 [negative mode], p-value < 0.00001).

Based on the extracted metabolites, the most contributing metabolite feature in both positive and negative modes was m/z 261.1414 [M + H]^+^ or 260.1360 (neutral mass after adduct deconvolution). This metabolite was tentatively identified as L-gamma-glutamyl-L-(iso)leucine. The intensity of this metabolite was found to be significantly correlated with storage time (R^2^ = 0.6378 [positive mode], R^2^ = 0.7893 [negative mode], p-value < 0.00001) (Fig. 6). This metabolite was also the highest ranked based on Hotelling’s *T*^2^ in MEBA in both positive and negative modes and was found to significantly change over time based on ANOVA (p = 1.26 × 10^−16^ [positive mode], p = 7.78 × 10^−25^ [negative mode]).

## Discussion

As metabolomics is rapidly expanding, thanks to developments in instrumentation and data analysis tools, several mechanisms previously unknown in certain diseases are now slowly being uncovered. This therefore provides an opportunity for scientists to look back at diseases that are currently being neglected, and yet still affecting millions of lives. Metabolomics, as appropriately titled in a 2015 paper “enter the battle against the neglected tropical diseases”^4^, is now being used as an important tool in studying severe acute malnutrition in children^17,18^ and many other diseases in low-resource areas^4^. However, although one must aim to achieve the highest level of quality, the storage and cold-chain logistical requirements of samples for metabolomics analysis is sometimes limiting the opportunity for this technique to be used in applications where the source of the biological material has limited resources. Unless the study site has a fully equipped laboratory and/storage facility, ensuring optimal freezing conditions of the samples could be daunting.

The effect of storage temperature on the stability of the plasma metabolome has already been described in the literature using different analytical platforms, such as gas chromatography – MS^19^, capillary electrophoresis – MS^20^, nuclear magnetic resonance (NMR) spectrometry^3^, and LC-MS^1,2,21^. According to these findings, plasma samples suited for metabolomics analyses should be stored at ≤−80 °C for long-term storage due to instability of many of the metabolites in plasma. In fact, the European Prospective Investigation into Cancer and Nutrition (EPIC) consortium in 2005 recommended the storage of plasma in liquid nitrogen for optimal preservation of metabolites, including rapidly degrading metabolites such as vitamin C^22^. Although liquid nitrogen or dry ice can be procured in many capital cities even in low-resource countries, delivering a constant supply of liquid nitrogen and dry ice cannot always be guaranteed, especially when the study site is very distant from a bigger city. Further, local flights often restrict the transport of these materials on-board. Also, installation of a −80 °C freezer could be a problem in low-resource settings where supply of electricity could be an issue. Therefore it is very interesting to determine whether significant deterioration of the plasma metabolome occurs upon temporary storage under sub-optimal conditions, such as −20 °C which is almost equivalent to a domestic freezer that can be easily maintained. Also, several passive cold chain procedures can sustain a temperature of −20 °C for a few days in case of power interruptions or while awaiting transfer to a −80 °C freezer in a more equipped laboratory.

Several papers have already reported that storage of plasma until 1 week at −20 °C is acceptable as no significant deterioration of the plasma metabolome was detected at this condition^2,3^. However, storage for up to 1 month at −20 °C causes deterioration of the plasma metabolome which could render it unfit for further metabolomics analyses^2,3^. While this information is very substantial, the amount of time between 1 week and 1 month could already dictate whether or not it is feasible to collect samples from low-resource areas. It is therefore important for us to estimate how long within the 1 month time frame is plasma still stable and fit for metabolomics analysis. Therefore, we have conducted a more closely timed experiment to assess the storage stability of the plasma metabolome under −20 °C, and to determine which biomarkers could be used to assess the stability of the plasma metabolome under these conditions.

Univariate analysis suggest that more metabolites were significantly different at 18 and 21 days in positive and negative modes, respectively. We however argue that a better analysis of metabolome stability should be to assess the individual change of these metabolites through time, which could not captured by univariate analysis. Thus in this paper, we report a time-resolved analysis of plasma metabolome deterioration under −20 °C using elastic net regularized regression implemented by the glmnet function in R. Compared to MEBA, where all metabolites are ranked based on their Hotelling’s *T*^2^ value, elastic net is a penalized regression model that includes a feature selection step, which extracts metabolite features that are predictive of storage time. The glmnet method used herein employed a Poisson regression model, which is a log-linear regression model to predict count data. Such method has been successfully used to predict the number of days a patient is likely to stay in a hospital using data from the electronic patient record system^23^. Similarly in this paper, we predicted the number of days the plasma has been stored at −20 °C based on the intensities of the metabolites detected by LC-high resolution MS. Glmnet has been employed in several studies in the field of genomics, especially associating gene expression and gene methylation with disease outcomes^10,24,25^. Glmnet, especially LASSO, has also been used in various metabolomics studies. For instance, this method has been used to predict the acamprosate treatment response in alcohol-dependent subjects based on metabolomics profile and clinical data^26^, and the age and sex of participants of the Karlsruhe Metabolomics and Nutrition (KarMeN) study based on their metabolomic profile^27^. To the best of our knowledge, this is the first report using elastic net on a time-series data for metabolomics analysis.

The elastic net model produced a highly predictive model with significantly high correlations between the actual storage time and the prediction in both training and test sets. The most contributing metabolite feature in both positive and negative modes was tentatively identified as L-gamma-glutamyl-L-(iso)leucine based on database comparison to the Human Metabolome Database (HMDB)^28^. This coincided with the results of MEBA and ANOVA, which supports the validity of the generated models. According to the HMDB, L-gamma-glutamyl-L-(iso)leucine is a proteolytic breakdown product of larger proteins. This metabolite is a plasma/serum biomarker for non-alcoholic fatty liver disease and non-alcoholic steatohepatitis^29^ and has been reported to increase in plasma of obese patients after consumption of whey isolate^30^. As this metabolite is a protein breakdown product, it is not surprising that the level of this metabolite is increasing with increasing storage time potentially due to the residual proteolytic activity that is not quenched at −20 °C.

As MEBA ranks all metabolites based on their Hotelling’s *T*^2^ value, which is indicative of the degree of their temporal changes, there are no absolute criteria on which Hotelling’s *T*^2^ value and/or rank signify that certain metabolites are considered to be of interest. For instance, in a paper studying the change in concentration of a potential biomarker for hepatocarcinoma over time, the top 30 metabolites with the highest Hotelling’s *T*^2^ value were considered as the final feature subset^31^. On the other hand, a study on urine metabolomic analysis to detect metabolites associated with the development of contrast induced nephropathy considered the top 4 metabolites based on Hotelling’s *T*^2^ values to be of interest^32^. Furthermore, a time-resolved metabolomics analysis of individual differences during the early stage of lipopolysaccharide-treated rats extracted 15 metabolites out of 60 identified metabolites as metabolites of interest based on Hotelling’s *T*^2^ rank^33^. In contrary, the proposed method in this paper is able to objectively distinguish which metabolites are changing across the time series and their temporal changes can be ranked based on the absolute value of the model coefficients.

Although we have pointed the advantages of using this approach, the method proposed in this paper applies only to one-sample problems (time-series data without additional independent variables) contrary to MEBA, which can resolve the interaction of time and another independent variable (i.e. treatment)^8^. Further research is required to develop a regularized regression model for datasets with additional independent variables. Furthermore, MEBA is designed to capture temporal changes even when there is no particular time-course patterns. In this paper, the assumption that degradation would elicit a time-course increase or decrease of certain metabolites was held. Hence, the utility of this model may only be best if a linear temporal pattern is expected.

Based on the extracted metabolite features using elastic net regularized regression, it can be seen in Fig. 5 that the metabolome appears to dramatically shift beginning around 12–15 days. Compared to previous data in the literature which suggests that 1 week storage at −20 °C is acceptable, our results show that plasma metabolome may still be used up to 2 weeks with very minimal changes in the profile. However, it is also noteworthy that the number of metabolites changing within the 1-month time frame are very minimal, which may suggest that plasma may in in fact be relatively stable at −20 °C for up to 1 month. This give more opportunity for field scientists to plan a strategic logistics cold-chain to obtain samples from low-resource areas for subsequent metabolomics analysis.

## Materials and Methods

### Plasma preparation and storage

Plasma samples were obtained from blood donors of the local blood bank (Ghent, Belgium) who have provided a signed informed open consent for the use of their blood for research purposes. Blood samples from 4 healthy donors were obtained in January 2017, immediately processed into plasma and blast frozen at −30 °C on-site and delivered to the lab. Plasma samples were then thawed in ice in the lab for further processing. As this study focused on the storage stability of plasma and not on the inter-individual variability, plasma samples from the four patients were pooled to generate a plasma reference material. This plasma mixture was used in lieu of (and to replicate) the NIST SRM 1950, which is expensive and offered in limited supply. The plasma mixture was then pipetted into 500 µL aliquots in 1.5 mL Eppendorf tubes. This research, including the methods performed herein, were approved by the Ghent University Commission for Medical Ethics (file number: EC/2017/0095) and are in accordance with its guidelines and regulations.

For the storage stability analysis, 100 tubes were immediately frozen at −20 °C while 10 samples were immediately frozen at −80 °C, which served as control (Time 0). Ten tubes were then transferred to −80 °C every 3 days. The long term stability of plasma at −80 °C has been demonstrated and thus it is inferred that the control sample would remain stable during the entire duration of the storage test^2^. Further, upon transfer of samples from −20 °C to −80 °C, degradation would most likely retard, thus keeping the current state of the metabolome stable until analysis.

### Metabolite extraction

At day 30, all plasma samples were thawed in ice for metabolite extraction. Plasma metabolites were extracted using ice-cold methanol pre-mixed with internal standards. To each 500 µL plasma aliquot, 1 mL of extraction solvent was added followed by rigorous vortexing and storage at −20 °C for an hour to allow protein precipitation. Thereafter, the tubes were centrifuged at 13.000 × g for 10 minutes at 4 °C. The supernatant (750 µL) was transferred to a glass tube and dried using a gentle stream of nitrogen at <10 °C. The dried pellets were reconstituted with 10% acetonitrile containing 0.1% formic acid and subjected to liquid chromatography-mass spectrometry analysis.

An aliquot of 10 µL was taken per sample and added together. This new sample served as a QC sample. A dilution series of the QC sample was made by diluting the QC sample in 0.1% formic acid at 1:1, 1:5; 1:10, and 1:50 (QC sample:water).

### Metabolite profiling

Chromatographic separation was performed using a Waters Acquity UPLC I-class FTN system (Waters, Manchester, UK) using both mobile phase solvent and flow rate gradients as suggested by Barri *et al*.^13^ with modifications. Mobile phase and flow rate gradients were adjusted between positive and negative modes to allow better peak separation within each ionization mode, as depicted in Table 2. The mobile phases consisted of 0.1% formic acid (A) and 0.1% formic acid in acetonitrile (B). Peaks were separated using an Acquity UPLC HSS T3, 100 Å, 1.8 µm, 1 mm × 100 mm column. Injection volume was 5 µL and the column was maintained at 50 °C.

The LC flow was directed to a Waters Synapt G2S*i* high resolution mass spectrometer (Waters, Manchester, UK) via electrospray ionization (ESI) in both positive and negative modes. Ionization capillary voltage was set at 2.75 kV for positive mode and 2.20 for negative mode. Source and desolvation temperatures were 150 °C and 500 °C, respectively. Cone and desolvation gas flow rates were 20 L h^−1^ and 600 L h^−1^. Mass range was 50–1000 Da and a scan speed of 0.1 s was applied in MS^E^ centroid resolution mode. MS^E^ collision energy was a ramp of 10–30 V for both positive and negative modes. Prior to mass acquisition, the mass spectrometer was calibrated using a mixture of sodium formate adducts. Internal calibration and online exact mass correction was applied using leucine-enkephalin (200 pg µL^−1^), which was infused to the MS every 10 seconds during acquisition at a flow rate of 20 µL min^−1^.

A blank sample (100% acetonitrile) was injected 10 times at the beginning of the analysis to condition the column. Then, the QC sample and dilutions of the QC sample were injected to the LC-MS. Blank and QC sample were re-injected after every 10 experimental samples in the run. Samples were analyzed in a random order.

### Data preprocessing and MEBA analysis

Data acquisition was monitored using MassLynx v1.7 (Waters). After data acquisition, data was imported to Progenesis QI (Nonlinear Dynamics, Newcastle, UK) for data pre-processing, including peak picking and deconvolution, and peak alignment using default settings from retention times 0 to 8.5 minutes. Thereafter, the data was exported to Excel (Microsoft) for data inspection and filtering. First, zero values were replaced with blank (empty cells). Features found in the QC samples that did not respond to dilution (R^2^ > 0.5; dilution factor versus feature intensities) were considered background noise and thus removed from the dataset. Then, features of the QC samples whose coefficient of variation were greater than 30 (%CV > 30) were also removed. Finally, features from the entire dataset which had more than 20% missing values were removed.

Univariate analysis and MEBA were performed using the time-series analysis tool in MetaboAnalyst^8,9^. Missing values were imputed using the K-Nearest Neighbor (KNN) algorithm and the data was normalized to constant sum and transformed using the generalized log transformation and autoscaling. One-way repeated measures ANOVA was used to determine features that significantly changed across the time series. Multiple testing correction was done using Benjamini-Hochberg correction^34^ at false discovery rate (FDR) p < 0.05. MEBA on the other hand was used to rank the features according to the differences in their temporal profiles based on the Hotelling’s *T*^2^ value, such that features with higher value are those that have higher time-course changes.

### Elastic net regularized Poisson regression

The pre-processed data was then used to build an elastic net regularized regression model using the glmnet package^35^ in R^36^. Elastic net is a generalized linear model that operates as a mix of ridge regression and LASSO, which was specifically designed to overcome issues of large variable number (metabolite features) and small sample size. Poisson regression on the other hand is used to model count data. In this case, we used the Poisson regression to model the number of storage days based on the metabolomic profile of each plasma sample. The log-likelihood function is given by Equation 1^35^:

1 $$l(\beta |X,Y)=\,\sum _{i=1}^{N}(yi({\beta }_{0}+\beta ^{\prime} {x}_{i})-{e}^{{\beta }_{0}+{\beta }^{T}{x}_{i}}.$$

where the elastic-net penalty is defined as Equation 2

2 $$\mathop{{\rm{\min }}}\limits_{{\beta }_{0},\beta }-\frac{1}{N}l(\beta |X,Y)+\lambda ((1-\alpha )\sum _{i=1}^{N}{\beta }_{i}^{2}/2+a\sum _{i=1}^{N}|{\beta }_{i}|\,)$$

where β_0_ and β are the coefficients of the linear model and *N* is the number of samples. The contribution of ridge regression and LASSO to model generation can be adjusted by adjusting the elastic net mixing parameter α, with range α ∈ [0, 1]. α = 1 is the LASSO and α = 0 is for ridge regression. Values in between adjust the contribution of ridge regression and LASSO α = 1 − ε for some ε < 0.5 performs much like LASSO while α = 1 − ε for some ε > 0.5 performs much like ridge regression. α = 0.5 equally imposes LASSO and ridge regression on the model. Before the data was passed to glment, the data were centered to zero and hence glmnet’s *standardize* option was set to *false* (standardize = FALSE).

The parameter λ is the tuning parameter (λ ≥ 0) which controls the strength of the shrinkage of the variables (metabolite features) and is optimized by cross validation. A leave-one-out cross-validation was used to determine the optimal value of regularization parameter λ based on both the minimum mean squared error (MSE) and minimum MSE + 1 standard error (SE) of the minimum MSE using the “cv.glmnet” function. The optimal λ value was then used for feature selection and model generation. Since the model aims to predict the number of days the plasma samples are in storage based on the metabolome profile, the data was fit in a Poisson regression model (family = “poisson”) and the loss to use for cross-validation was set to “type.measure = deviance”.

To build the model, the data was initially split into training and test sets using the createDataPartition function in the caret package^37^. The data was randomly split into training set (75% of the data set) and test set (25%). Model generation was performed using the training set and the test set served as an external validation of the model. The model was assessed by calculating the root mean squared error (RMSE) of the training set and root mean squared error of prediction (RMSEP) of the test set, and the correlation between the number of days the plasma was stored at −20 °C and the predicted storage days using Pearson correlation (R^2^ > 0.8, p < 0.05) in both training and test sets. Features extracted from the elastic net Poisson model were ranked based on the absolute values of the model coefficients, such that higher values indicate more important contribution to the model. The extracted features were examined using a heat map and principal components analysis (PCA) in MetaboAnalyst and R. Unlike conventional regression modelling, the penalized regression model employed in this paper does not assign a statistical significance (p) value for the extracted metabolite features. Instead glmnet includes bootstrapped cross-validation for tuning and selecting the optimal λ, as well as the selection of the penalization parameter (α), which penalizes the metabolite feature coefficients. Metabolite features whose penalized coefficients are > 0 are retained in the overall model in the elastic-net framework^24^.

## Conclusion

In this paper, we have demonstrated the use of elastic net regularized regression to study the degradation of the plasma metabolome under a sub-optimal freezing condition. Using this method, metabolites were ranked based on their temporal profile while simultaneously discarding metabolites whose temporal changes were negligible, allowing a more focused and objective selection of interesting metabolites. Furthermore, our data suggests that the plasma metabolome is relatively stable at −20 °C with few metabolites drastically changing until 1 month. However, a good compromise between storage temperature and plasma stability is seen at 12–15 days storage. This provides more information for logisticians to plan sampling and storage procedures for collecting biological samples from low-resource areas.
