# Author Correction: Elastic net regularized regression for time-series analysis of plasma metabolome stability under sub-optimal freezing condition

**DOI:** 10.1038/s41598-018-27563-2

**Published:** 2018-06-15

**Authors:** Gerard Bryan Gonzales, Sarah De Saeger

**Affiliations:** 1 Gastroenterology and Hepatology, Department of Internal Medicine, Faculty of Medicine and Health Sciences, Ghent University, C. Heymanslaan 10, 9000 Ghent, Belgium; 2 Laboratory of Food Analysis, Department of Bioanalysis, Faculty of Pharmaceutical Sciences, Ghent University, Ottergemsesteenweg 460, 9000 Ghent, Belgium

Correction to: *Scientific Reports* 10.1038/s41598-018-21851-7, published online 26 February 2018

This Article contains an error in Figure 5, where the Negative mode heat map is a duplication of the Positive mode heat map. The correct Figure 5 appears below as Figure 1.

**Figure 1:**
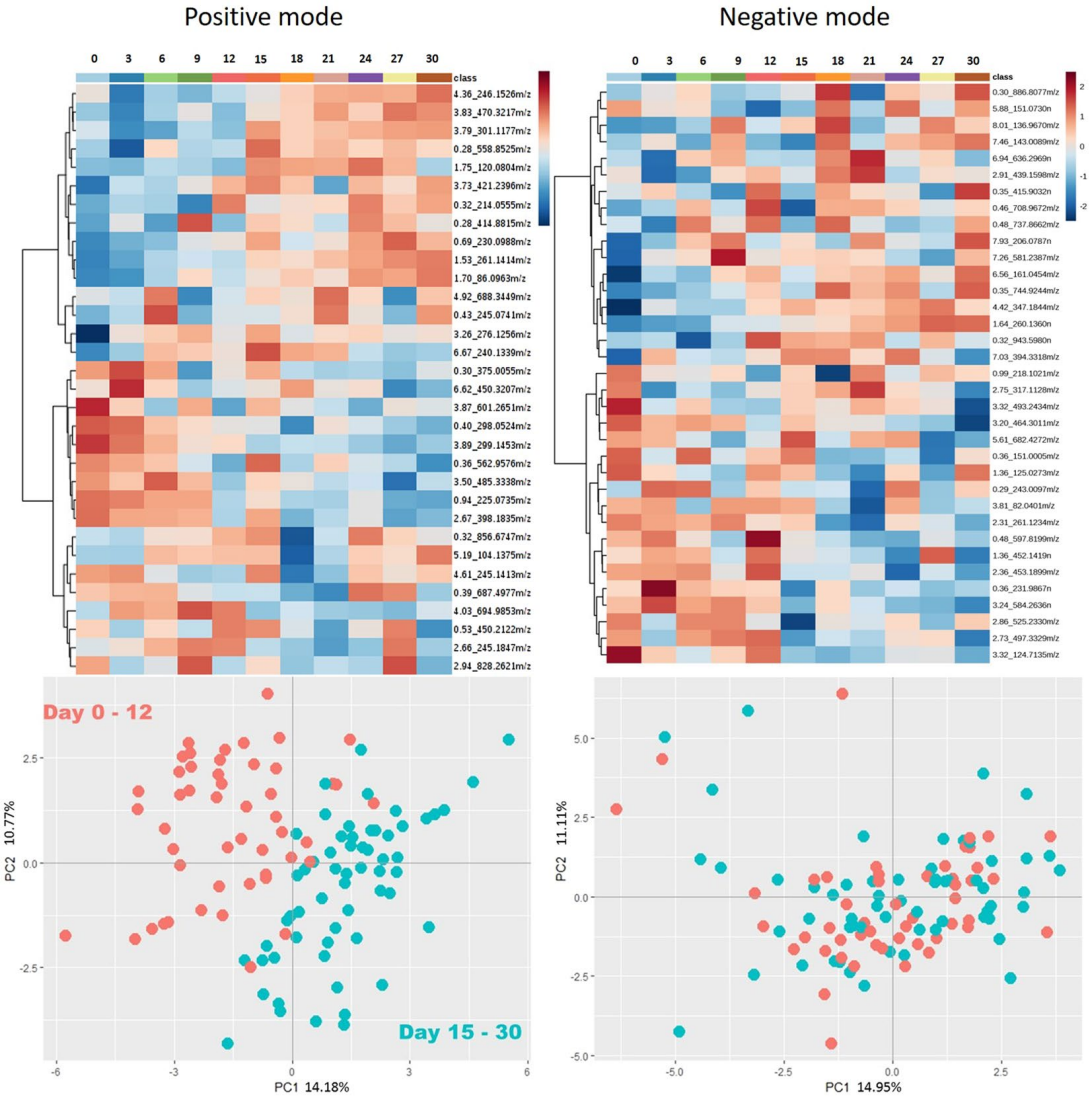
Heat maps (top) and PCA plots (bottom) of metabolites extracted by the elastic net regularized Poisson regression (glmnet) model over the time series in both positive and negative modes. The elastic net model extracted 32 metabolites of interest in both ionization modes.

